# Reaping the benefits of digital transformation through Public-Private Partnership: A service ecosystem view applied to healthcare

**DOI:** 10.1007/s43508-022-00056-9

**Published:** 2022-11-16

**Authors:** Elena Casprini, Rocco Palumbo

**Affiliations:** 1 Department of Business and Law, University of Siena, Siena, Italy; 2 Department of Management and Law, University of Rome “Tor Vergata”, Via Columbia, No. 2, 00133 Rome, Italy

**Keywords:** Public–Private Partnership, Robotization, Automation, Healthcare, Knowledge-based service ecosystem

## Abstract

The ongoing digital transformation ushers unprecedented challenges for publicly owned healthcare organizations. Collaborative governance models, such as Public Private Partnerships (PPPs), advance their readiness to address such challenges, paving the way for the establishment of a viable service ecosystem. However, little is known about how PPPs enhance the publicly owned healthcare organizations’ ability to thrive amidst the digital transformation. The article investigates this issue, drawing on the exploratory case of “Lab@AOR”, a PPP established between Loccioni and the University Hospital of Marche (Italy) which focused on the robotization of a critical component of healthcare services’ delivery. Three ingredients have been found to nurture the PPP’s cohesiveness and success: (1) the alignment between the public partner’s needs and the private partner’s competences, (2) knowledge contamination, and (3) the adoption of patient-centeredness as the inspiring principle of the collaboration. The PPP represents an initial step of the transition towards a service ecosystem, entailing a fully-fledged partners’ integration for value co-generation.

## Introduction

Digital transformation and the advent of robotization reshape all spheres of economic activity (Brodny & Tutak, 2021), setting the ground for new business opportunities (Kokshagina, 2021). Furthermore, they generate unprecedented management challenges (Czarniawska & Joerges, 2020), enacting disruptions which put the organizational capability to generate value under stress (Kane, 2019). This is especially true in the healthcare sector, where digitalization and innovative Information and Communication Technologies (ICTs) propel a reconfiguration of management practices (Ciasullo et al., 2022), reframing health services’ design and delivery (Odone, 2021). Alongside altering organizational processes, the digital turn creates wicked issues for healthcare institutions. It paves the way for technocentricity in the delivery of care, overlooking the human nature of health services (Howard, 2021). Besides, it yields a high-tech, but low human-touch service environment, producing intricate ethical concerns for healthcare organizations (Saurabh et al., 2022). Finally, yet importantly, it produces sources of stress and strain at work, exacerbating time pressures and job insecurity (Palumbo & Cavallone, 2022). If not properly addressed, these issues are expected to impair the transition towards a 4.0 approach in healthcare (Cavallone & Palumbo, 2020).

The limited capability of healthcare organizations to cope with these challenges determines resistances to change and prevent the smooth digital transformation of health service delivery (Landaeta et al., 2008). Healthcare organizations rely on different approaches to overcome resistances to change, endeavouring to take advantage of digital technologies (Agarwal et al., 2010) and achieve an improvement of health services’ quality and effectiveness (Sandhu, 2020). Among them, Public–Private Partnerships (PPPs) have attracted the growing interest of scholars and practitioners (*e.g.*, Casady & Baxter, 2022; Ganapathy & Reddy, 2021; Ma et al., 2021). PPPs entail “…a sustained, collaborative effort between the public and private sectors in which each contributes to the planning and resources needed to accomplish a mutual objective” (Spielman & von Grebmer, 2004: p. 40). PPPs have manifold advantages (*e.g.*, Greve et al., 2022; Kosycarz et al., 2019; Wright et al., 2019), such as boosting the organizational attractiveness towards differentiated sources of funding (Lim, 2004) and enriching available resources and competencies (Ganapathy et al., 2021), thus setting the conditions for a smooth digital transformation of healthcare organizations. Literature has also warned of the shortcomings of PPPs, which might determine a loss of public control over value creation, as well as accountability issues (Engel et al., 2014).

To the best of the authors’ knowledge, there is limited understanding of the factors that underpin the PPPs’ effectiveness in fostering the digital transformation of publicly owned healthcare organizations. The article intends to push forward what we know about the determinants of PPPs’ success in healthcare. It collects evidence of the implications of PPPs on healthcare organizations’ readiness to enact a shift towards automation and robotization, winning resistances to change and nurturing the establishment of a service ecosystem which fosters continuous innovation and sustains the development of the PPP. More specifically, the following research question triggered this study:

- *R.Q.*: How does the development of a successful PPP unfold in the healthcare context to boost digital transformation?

The paper is organized as follows. Section 2 presents the conceptual background against which this study was established, shedding light on the role of PPPs for fostering innovation and enacting viable service ecosystems. Next, the study methodology is presented in Sect. 3. Section 4 reports the study findings, envisaging the steps of the PPP’s development. The results are critically discussed in Sect. 5, which inspires the conceptual and practical implications of this research, as reported in Sect. 6.

## Conceptual background

PPPs’ role in healthcare is controversial, presenting bright and dark sides (Torchia et al., 2015). PPPs have been largely argued to enhance the functioning of healthcare organizations and improve the delivery of health services (Baliga et al., 2016; Jack & Phillips, 1993; Kumar, 2003). As previously anticipated, PPPs are risk sharing relationships between public sector entities and private sector companies, which are aimed at conjoining the partners’ efforts to achieve a desired outcome in terms of public value generation (Field & Peck, 2003). Drawing on Bath (2000: p. 4706), PPPs “…in the health sector can bring needed resources while also taking care that the vulnerable groups (…) have access to health facilities”, advancing the value generation capability of publicly owned healthcare institutions. PPPs are conducive to establishing reliable infrastructures and enabling the timely provision of high-quality health services (Sekhri et al., 2011). For this to happen, they combine the values which inspire public action with the entrepreneurial orientation and managerial proficiency of partners operating in the private realm (Roehrich et al., 2014). Harmonizing public and private efforts, PPPs drive the gradual build-up of a service ecosystem, which relies on value co-creation to achieve long-term viability (Brodie et al., 2021). From this standpoint, PPPs bring with themselves several advantages, augmenting the partners’ innovation capability, overcoming financial constraints, and tackling problems of integration (Sharma & Seth, 2011), which are especially common in the healthcare sector (Barlow et al., 2013).

Nevertheless, PPPs enact unprecedented management and organizational challenges (Adamou et al., 2021). Inability to timely and effectively manage them expands the dark side of PPPs. McQuaid (2000: p. 22) summarizes the potential disadvantages of PPP in “…unclear goals, resource costs, unequal power, cliques usurping power, impacts upon other ‘mainstream’ services, differences in philosophy between partners and organizational problems”. Moreover, PPPs determine increased managerial burdens for public sector partners and private entities involved in the collaboration. Pratici and Singer (2021) identified three factors adding to the management complexity of PPPs in healthcare, namely performance measurement, compensation, and risk management. Another important problem of PPPs is related to their transaction costs: even if opportunism may be controlled via formal (*e.g.*, detailed contracts between partners) and relational tools (*e.g.*, codes of conduct and norms of behaviour), finding an alignment between governance mechanisms and partners’ needs is not easy (Maurya & Srivastava, 2019). Lastly, entailing a contamination of the public interest with private needs, PPPs are thought to undermine the equitable provision of health services, reducing the effectiveness of healthcare organizations (Hellowell & Pollock, 2009).

Tailored organizational and management efforts are needed to catalyse the *pros* and fix the *cons* of PPPs, establishing profitable collaborations to enhance the functioning of health systems (Bedenkov et al., 2020). However, only few attempts have been made to examine how PPPs may foster the digital transformation of healthcare organizations (Kosycarz et al., 2019). Previous research highlighted the importance of interactions across multidisciplinary teams and professional groups (Scott et al., 2018) and the need for expanding the reach of collaborative networks outside the organizational boundaries of healthcare institutions (Baudin et al., 2020; Enkel, 2020). Notwithstanding, scholars are not consistent in unravelling the strategic, organizational, and management processes that are conducive to the establishment of effective PPPs fostering digital transformation in healthcare (Furtner et al., 2021). This is a major gap in the scientific knowledge, since previous studies have stressed that embracing an ecosystem view which sustains public–private interactions is essential to boost the digital transformation of healthcare organizations (Santarsiero et al., 2022).

In spite of these considerations, PPPs have been argued to play a critical role in the development of a viable service ecosystem engendering “…the coevolution and the context of interactions among stakeholders in the value co-creation process, emphasising the various relationships and roles assumed by network members and increasing the efficiency of operant resource” (Petrescu, 2019: p. 1748). Moving from the integrative framework arranged by Osborne et al. (2022), we zoom in on PPPs, considering them as a peculiar form of collaborative governance. Collaborative governance falls in the meso-level dimension of public service ecosystems, representing the habitat within which relevant stakeholders participate in the process of public value generation (Osborne, 2021). More specifically, the meso-level “…concerns the service level of public service delivery” (Osborne et al., 2022: p. 639). It looks at value in production fostered by co-design and co-production initiatives which are boosted by manifold interactions among different stakeholders. Embracing this perspective, our paper aims at illuminating the complex dynamics leading to the establishment and the implementation of a PPP intended to facilitate the reconfiguration of health services’ delivery in a digital perspective. For this purpose, we investigate an exploratory case delving into how a PPP sets the conditions for the development of a viable public service ecosystem.

## Methods

### Study strategy and design

To understand how PPPs sustain the process of digital transformation in healthcare and set the ground for the establishment of a service ecosystem, we adopted an exploratory case study, which enabled us “…to inductively generate, rather than deductively confirm, insights regarding the phenomenon of interest” (Ogawa & Malen, 1991: p. 271). This methodology is recommended when the phenomenon under investigation is in its real-life, natural context (Yin, 1994). A recent literature review pointed out that the case study approach is the preferred method to analyse the critical success factors of PPPs (Osei-Kyei & Chan, 2015). Previous studies have used this approach to investigate the implementation of PPPs in the healthcare sector: attention has been paid to the ethical challenges generated by PPPs (Hebson et al., 2003), the hybridization of public value generation processes (Villani et al., 2017), and the implementation of global partnership for health promotion (Patnaik et al., 2020). As supported by these references, the case study method can be considered appropriate for answering our research question.

The case was selected based on multiple reasons. The first motive relies on its international relevance. As explained in the following section, “Lab@AOR” can be conceived of as an example of a successful PPP which, although originated at local level, has soon expanded internationally, operating in three different Continents (*e.g.*, Soumoy & Hecq, 2019). The second motive is represented by the fact that the partners initially involved in the PPP ensured us with a large access to data. One of the authors had multiple professional exchanges with the private partner over the last decade. Hence, a fully-fledged understanding of the case was possible, permitting us to straightforwardly collect relevant evidence to answer the research question. The third reason is linked to the “Lab@AOR” focus on the creation of a knowledge-based service ecosystem, where innovative models of care have been introduced to foster the digital transformation of healthcare according to a patient-centered perspective (Palumbo et al., 2017).

### Data collection and analysis

As recommended by the scientific literature (*e.g.,* Chetty, 1996; Yin, 1994), multiple sources were used to collect data, relying on primary and secondary sources of information. As far as primary data are concerned, 6 interviews have been conducted with 6 key informants over the last 4 years. We also used information obtained from direct observations conducted by one of the authors for about 2 days in 2019 and by both the authors for about 3 days in July 2022 within Loccioni and the University Hospital of Marche. Loccioni provided the authors with internal documents, which delivered in-depth information about the topics being investigated. As far as secondary data sources are concerned, the authors relied upon two book chapters and two scientific papers. Besides, 19 new press releases were collected from the Lexis Nexis® database. Finally, the authors collected evidence from videos available on YouTube. A summary of data used for the purpose of this study is available in Table 1.

**Table 1:** An overview of primary and secondary data

Type of sources	Source	Date	Length
1 Interview	Claudio Loccioni	2019	56 min
1 Interview	Two employees involved in transversal teams’ operations	2019	45 min
1 Interview	Claudio Loccioni	2022	90 min
3 Interviews	Three senior managers of the University Hospital of Marche	2022	110 min
Publications	Book chapter	2019	(Casprini, 2019)
	Paper	2019	(Ombrosi et al., 2019a)
	Paper	2017	(Casprini et al., 2017)
	Book chapter	2019	(Ombrosi et al., 2019b)
New press releases	Lexis Nexis ®	2022	19 news press releases
Videos	Share/Care with Vincenzo Moretti—Hospital Pharmacy (https://www.youtube.com/watch?v=iaas6Z6fk7Y)	03/12/2014	86 min
	Gruppo Loccioni—Apoteca (https://www.youtube.com/watch?v=bLv_18wePHY)	29/10/2013	4:40 min
	Safe and integrated onco-hematology workflow at Ancona University Hospital (https://www.youtube.com/watch?v=RIhIGZjPyqc)	18/04/2014	2:08 min
	Share/Care with Pietro Leoni—Hematology (https://www.youtube.com/watch?v=8_PVf85TseQ)	05/02/2015	92 min
	APOTECA Chemotherapy Mixing—Cone Health Cancer Center (https://www.youtube.com/watch?v=DGenr_Qc3tU)	31/07/2018	1:15 min
	APOTECAchemo Doxorubicin Preparation (https://www.youtube.com/watch?v=a0SZt32KsZc)	07/02/2016	3:12 min
	APOTECACommunity—International meeting 2014 (https://www.youtube.com/watch?v=ek8_uPujI28)	06/10/2014	3:30 min

Data analysis was accomplished through three steps. First, an analysis of each of the two partners involved in “Lab@AOR” was conducted. This analysis allowed us to better understand the traits of each entity involved in the partnership, before concentrating the attention on the development of the PPP. Second, the authors investigated the PPP looking at the main events that characterized its evolution. Following a process study approach (Langley et al., 2013), the authors identified the following three main phases: (1) preparing the ground for partnering (pioneering stage), (2) nurturing collaborative exchanges between partners (exploitation stage), and (3) unleashing the potential of the PPP (harvesting stage). Third, the authors identified the key themes that have characterized the process of digital transformation within each phase. In doing so, the authors relied upon a coding procedure to systematize and arrange data (Miles et al., 2014). The pioneering stage is characterized by the attempt to exploit the partners’ distinctive bundle of resources, envisaging unprecedented opportunities through collaboration. The exploitation stage is rooted on the previous one and entails knowledge contamination and integration to boost the partners’ ability to overcome internal resistances and participate in value co-generation. The harvesting stage involves the assessment of value in production enabled by the partnership, as well as the identification of avenues for further development, which activate a new pioneering stage.

### The case experience

“Lab@AOR” resulted from the collaboration between a publicly owned hospital—the University Hospital of Marche—and a private company—Loccioni. The University Hospital of Marche (Azienda Ospedaliero Universitaria delle Marche—AOUM) has been established in 2003, resulting from the involvement of the Polytechnic University of Marche in the management of the former hospital of Ancona. It currently has 10 Departments and employees about 3,500 people, including more than 100 professors. The University Hospital of Marche is particularly focused on innovation and ICT solutions for e-Health. As an illustrative example, the Oncology Department is involved in the Molecular Tumor Board, where multidisciplinary teams aim at identifying a tumor genomic profile. This typifies the commitment of the University Hospital of Marche towards precision medicine.

Loccioni is a family firm located in Angeli di Rosora, a small-sized municipality nearby Jesi, in the Marche Region (Italy). The history of the company dates back to 1960s, when Enrico Loccioni, an electrician, and Graziella Rebichini, Enrico’s wife, decided to start their own business in the bucolic landscape of Angeli di Rosora. Over the years, the company has grown in terms of collaborators (as Loccioni refers to its employees), turnover, and markets served. Measurement is the core competence of the company. *Inter alia*, it has been applied to white appliances (*e.g.*, measurement of the number of vibrations of a washing machine) and the automotive industry (*e.g.*, measurement of cars’ emissions), as well as to healthcare (*e.g.*, robotization of health services’ delivery). Currently, the company has about 500 collaborators and a turnover of about 70 million euros. An open culture nurtures its development, prompting the company’s readiness to establish collaborations with private and public sector organizations.

## Findings

### Preparing the ground for partnering: the pioneering stage

The PPP embodied by “Lab@AOR” was set up in 2006. In its early stages, an asymmetric relationship tied the partners, which primarily cooperated to enhance the quality of critical health services delivered to vulnerable patients. The focus was put on a single phase of the healthcare treatment delivered to a particular category of users: the in-hospital preparation of medications obtained from the combination of hazardous components for oncological patients. The public partner, *i.e.*, the University Hospital of Marche, perceived a gap in its innovation capability and readiness to accomplish an organizational transition to accommodate the shift towards automation. As reported by a key informant of the hospital, “…it fell short in identifying a timely, feasible, and effective solution to improve the quality, the appropriateness, and the efficiency of hazardous intravenous compounding”, which represents a critical component of appropriate health services targeted to oncological patients. Alongside being a key ingredient of the recipe for high-quality health treatments, “…several challenges affected the implementation of this activity”. A small fluctuation in the composition of the drug might have major side effects on the patient, leading to a lethal outcome. Besides, the hazards faced by people involved in the preparation of the compound make this activity risky and unattractive to health professionals. Third, the potential waste of resources associated with traditional approaches to produce hazardous intravenous compounding undermines organizational efficiency. Lastly, specific attributes related to the individual clinical conditions should be taken into consideration for the preparation of a compound which is fully consistent with the illness and minimizes side effects. As consistently reported by the members of the senior management of the public partner, the hospital was inert to processes of change which modified the contents and the processes of healthcare delivery, enacting unprecedented challenges for healthcare professionals. The private partner, *i.e.*, Loccioni, was interested in testing and continuously improving APOTECAchemo, an innovative technological solution conceived by the company to enhance the process of hazardous medications’ preparation through digitalization and robotization. As argued by a member of the team in charge for the design and implementation of the technology, “…APOTECAchemo primarily consisted of a robotized system enabling the automation of the whole process of oncological medication preparation”, overcoming most organizational challenges and operational issues faced by healthcare institutions in accomplishing this activity. Drawing on the remarks of the senior managers of the company, even though Loccioni had strong technological proficiency and advanced capability in the field of measurement systems, it “…needed a field testing of APOTECAchemo to collect rich data and refine both the contents and the features” of the technological solution.

Hence, the PPP emerged as an inter-organizational collaboration which was intended to overcome two different expectations of the partners: the need for transitioning towards a digitalized model of compounds’ production perceived by the University Hospital of Marche and the need for testing innovative technologies into a real-life setting perceived by Loccioni. The conjunction of these two expectations was conducive to public value co-generation in terms of increased quality and appropriateness of care delivered to vulnerable patients, thus surpassing the sphere of interest of individual partners. Far from representing a mutual exchange between the partners, “Lab@AOR” was understood as a co-design laboratory, whose main purpose was to augment the innovation capability of both the organizations involved in the PPP. As one of Loccioni’s collaborators argued: “The Hospital needed to bring business culture within itself, and we needed to bring clinic culture within Loccioni, since we cannot think gathering all the specialized knowledge about healthcare delivery processes (…). To have a place where we can go, gather information, and deepen health-related topics: this is fundamental”.

In line with these considerations, people involved in the partnership endeavoured to build an inter-organizational identity which inspired their commitment to the “Lab@AOR” and fostered their active participation to initiatives implemented at the crossroad of the partners’ organizational boundaries. A distinguishing mission was assigned to the PPP, which—as reported in the institutional documents presenting the initiative—was framed as a “…collaborative arena co-generated by the University Hospital of Marche and Loccioni relying on the enabling role of technologies to foster a dialogue across disciplines and continuously improve the individual and collective wellbeing”. A vision was attached to the “Lab@AOR”, which was envisioned as a co-creating laboratory where professionals, scientists, and experts meet to share their knowledge, combine their competencies, and contaminate their perspectives to build a new healthcare system.

Consistently with the mission and vision attached to the PPP, a set of shared values was identified by the partners, which were acknowledged as the glue binding them to the fulfilment of inter-organizational activities. *Humanity* was set as the underpinning value of the PPP, whose focus is on the development of innovative technologies which possess a high human touch and do not desensitize health services’ delivery, despite automation and robotization. *Humanity* is merged with *openness*, with innovation being conceived of as the result of a continuous dialogue involving all the interlocutors who are directly or indirectly interested in the improvement of healthcare delivery processes in a perspective of viable value co-generation. For example, one of Loccioni’s collaborators maintained: “…there are ‘dialogue tables’ along different levels (…). We continuously exchange information: the chief physicians come here, and they tell us the challenges and the problems they are facing (…). Maybe, they would not end in any project, but this is dissemination”. *Exploration* identifies the key principle guiding individual and collective actions across the PPP. “Lab@AOR” is not simply aimed at solving extant problems. Rather, it shows a discovery orientation, highlighting new perspectives for improving the quality and the effectiveness of care. Lastly, *science and measure* express the PPP’s commitment to the generation of tangible impacts for people and, more in general, the community, through the enhancement of the appropriateness of care and the improvement of achievable health outcomes.

**Fig. 1:**
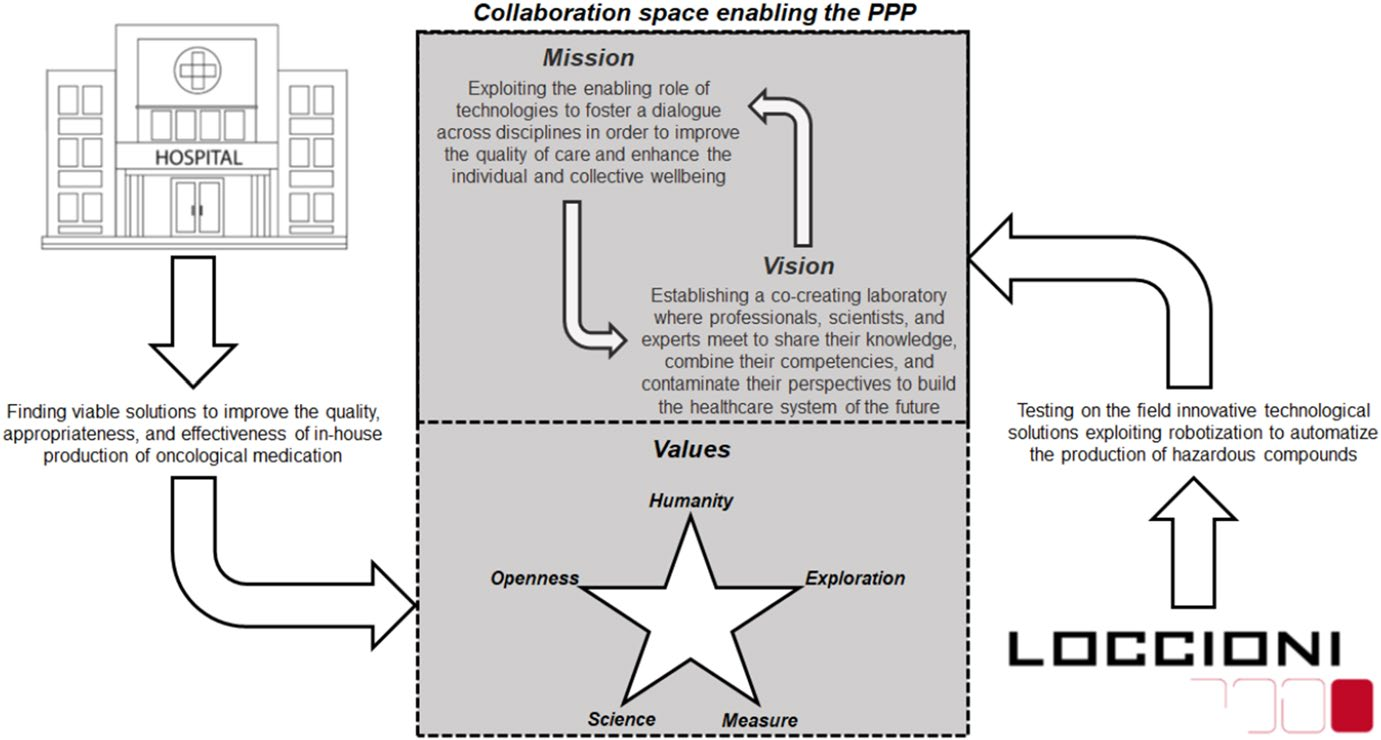
The origins of “Lab@AOR” as a PPP

Figure 1 graphically depicts the beginnings of the PPP, pinpointing the factors which triggered the collaboration between the partners and illustrating the values against which the University Hospital of Marche and Loccioni established their inter-organizational relationship.

### Nurturing collaborative exchanges between partners: the exploitation stage

The partners coalesced to adapt the original design of APOTECAchemo to the organizational challenges and management issues faced by healthcare institutions in arranging the in-hospital production of medications for oncological patients. The “Lab@AOR” hosted a common workplace, where transversal teams composed of members of the University Hospital of Marche and Loccioni met to refine the technology and make it consistent with the contingencies affecting the delivery of health services to people living with oncological diseases. Transversal teams consisted of physicians, nurses, pharmacists, as well as engineers and computer scientists. The teams’ operations were compliant with four guidelines as follows: (1) *transparent communication*: barriers to the exchange of information were removed, facilitating an open flow of ideas and perspectives; (2) *networking*: the teams were empowered to experiment and cope with issues preventing the implementation of the new technological solution in the production of oncological medications; (3) *continuous education*: people participating to the “Lab@AOR” were solicited to advance their capabilities and skills, especially through dialogue and knowledge contamination; and (4) *innovation orientation*: the focus was put on introducing unprecedented solutions, which were conducive to momentous improvements of health treatments’ quality.

This code of action enabled partners to push forward the contents of the PPP’s core technology, *i.e.*, the APOTECAchemo, and to find new opportunities for application of ideas conceived within and across transversal teams. The insights developed during the testing and experimentation of APOTECAchemo were further exploited to craft a new solution, APOTECAunit, which applied the discoveries achieved by the transversal teams to the robotized in-house production of sterile medication for non-oncological purposes. As argued by a key informant, this represented a sort of “…gemmation of the APOTECAchemo, expanding its applicability out of the context for which it was initially conceived”. Moreover, the PPP paved the way for the design of a digital tool, namely APOTECAps, supporting health professionals in the manual preparation of sterile injectable medications. This spill-over added to the contents of the technological solution which originally prompted the PPP, being conceived of as a “…device going beyond robotization and setting the conditions for an improved human–machine interaction”. It augments human action with the insights and feedback delivered by Information and Communication Technologies (ICTs), combining personalization and precision in the delivery of health treatments to patients. As described by one of Loccioni’s collaborators: “…there is a training period for people who approach the technology (…). Nevertheless, these technologies have been thought to be used by persons with no ‘technical’ expertise (…). It has been done a huge work at the beginning, also in terms of design (…). For example, how the software should look like, user-friendliness, which were the procedures and the workflows (…)”.

**Fig. 2:**
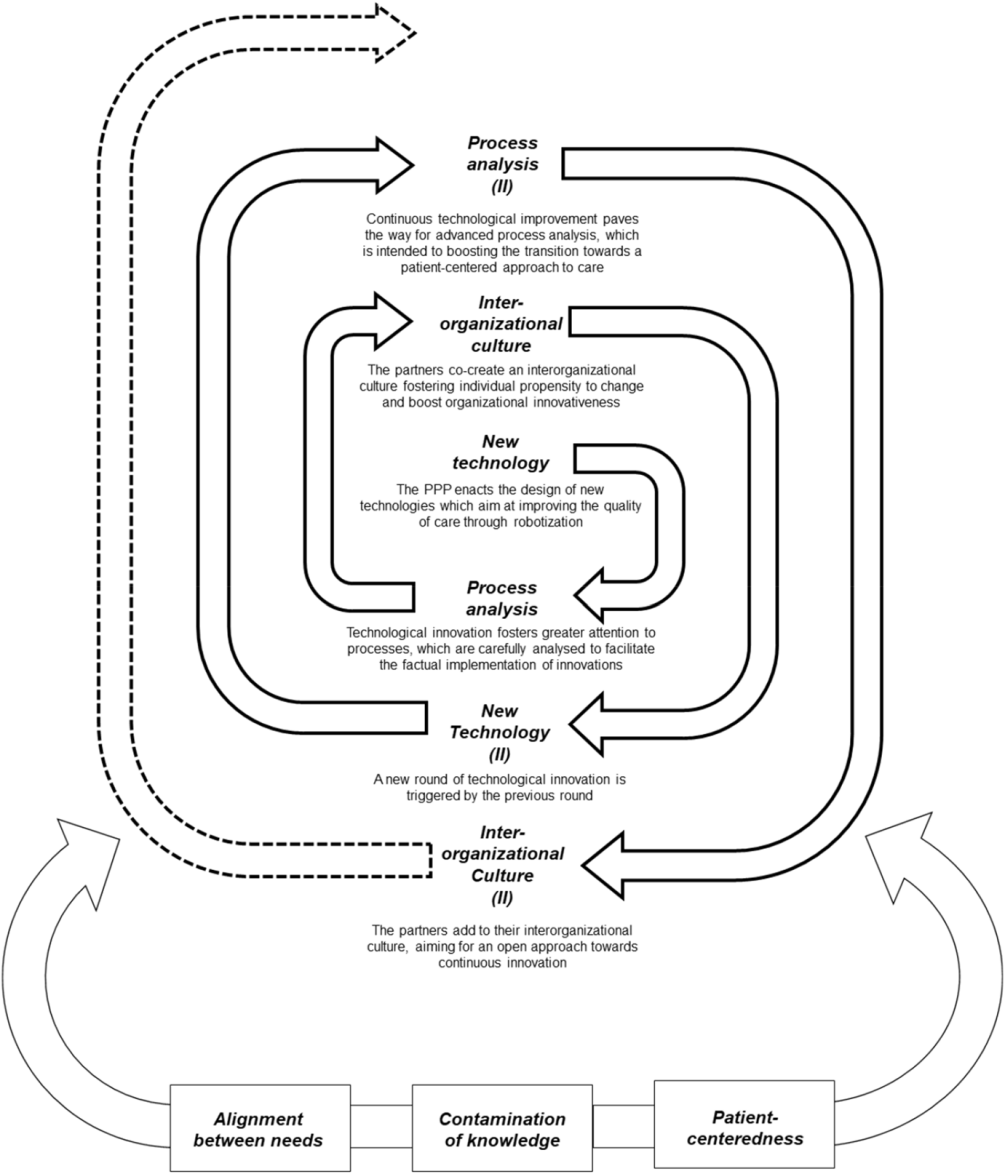
The steps of “Lab@AOR” development as an innovation-oriented PPP

As depicted in Fig. 2, the expansion of the PPP’s attention on innovative technological solutions blossoming from APOTECAchemo enacted a spiral of development, which can be articulated in the following three main steps: (1) new technology development, (2) process analysis, and (3) creation of an inter-organizational culture. The first step includes crafting new technologies, which boosted the early growth of the PPP. Such new technologies solicited the transversal teams to devote time and efforts to the analysis of healthcare processes, with the main purpose of envisioning new paths for enhancing the quality and effectiveness of care. The concern for process analysis brought the partners to reconfigure the original architecture of APOTECAchemo, enacting an APOTECA ecosystem, which can be considered as the second step. On the one hand, the APOTECAm@a software was introduced: it consists of a statistical package enabling a continuous and contextualized analysis of data. Through its reporting system, it provides a valuable support to the users of the APOTECA solutions, empowering them to carefully monitor the robotized activities. On the other hand, the APOTECAmanager is implemented as follows: it is designed as a management control system, which oversees the overall functioning of the APOTECA ecosystem and sustains the continuous improvement of automatized systems to produce medications obtained from hazardous and non-hazardous compounds, thus enhancing the timeliness and effectiveness of care. These different technological solutions are harmoniously integrated through a datacentre, which is named Agorà. Operationalizing an ecosystem view of the APOTECA solutions, Agorà pursues four main purposes: (1) improving the safe production of hazardous and non-hazardous medications through robotization; (2) increasing the organizational efficiency, reducing wastes and achieving a better allocation of available resources; (3) simplifying organizational procedures and practices; and (4) facilitating information access and advancing the individual ability to timely share evidence contributing to organizational process improvement.

The third step of the spiral regarded the creation of an inter-organizational culture, which was intended to win inertia and resistances preventing the partners from getting advantage of the innovative solutions conceived within the PPP. The culture permeating the inter-organizational activities of “Lab@AOR” was established on the founding principle of *patient-centeredness*. Although the focus was on the development of high-tech solutions to enhance the quality and effectiveness of care, improving the patients’ service experience represented the leading outcome inspiring the functioning of the PPP. This pivotal value was embedded within artifacts and symbols. Co-design and co-production were used as artifacts emphasizing the need for shifting towards patients’ empowerment and centrality throughout the healthcare delivery process. Symbolic action was encapsulated in informal meetings during which the members of the partners gathered to nurture the development of an inter-organizational culture facilitating the exchange of information and knowledge.

### Unleashing the potential of the PPP: the harvesting stage

**Fig. 3:**
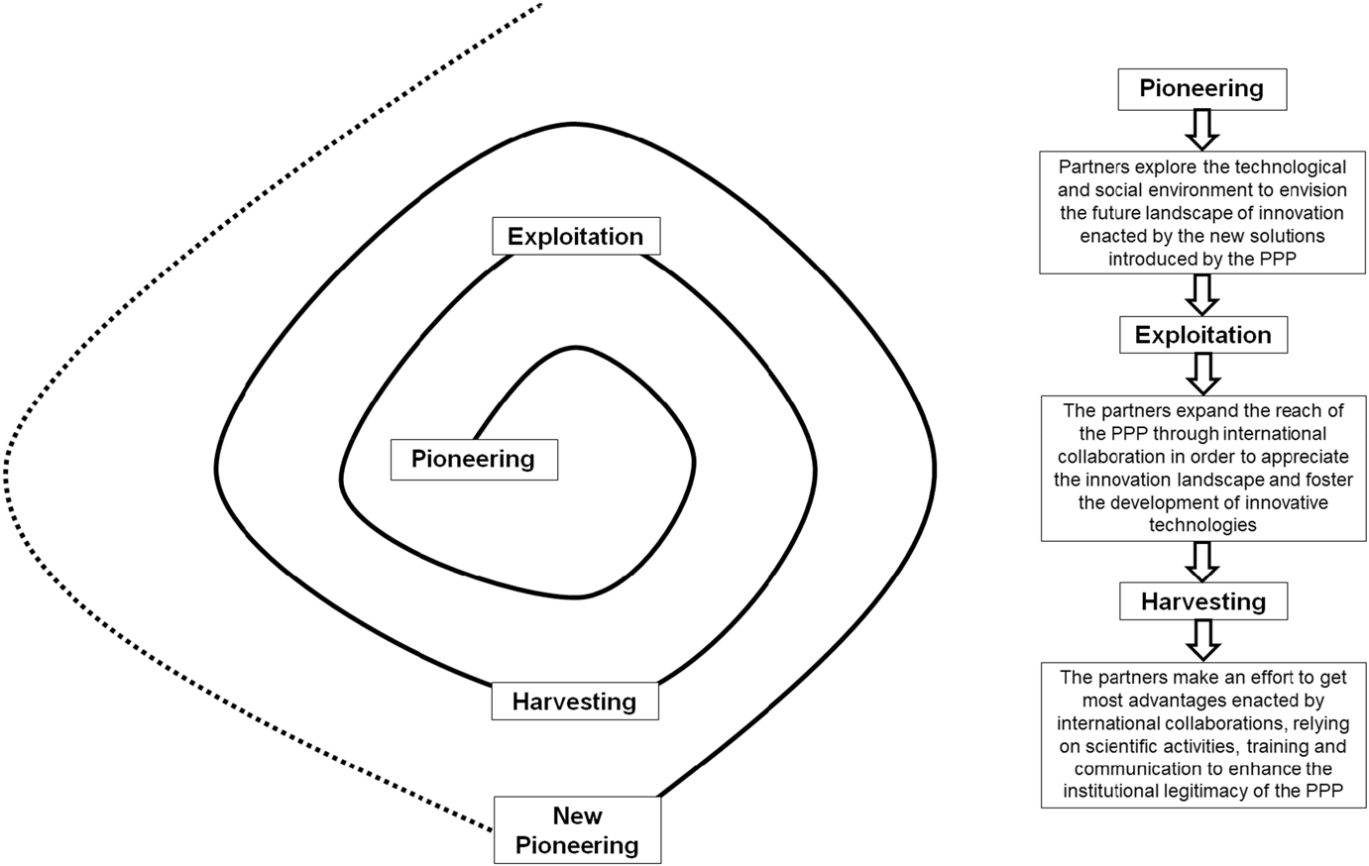
The international development of the PPP

Operating within the Italian context, the “Lab@AOR” pioneered in the development and implementation of a robotized system for the in-house preparation of hazardous and non-hazardous medications targeted to patients served in the hospital setting. The need for enhancing the quality and effectiveness of technological solutions embedded in the APOTECA ecosystem fostered the partners to expand the partnership’s reach, reinterpreting the PPP as an international community focused on getting advantage from digitalization and robotization for improving the quality of care and achieving patient-centeredness. As shown in Fig. 3, *pioneering*, which can be conceived of as the exploration stage of the PPP, was followed by an *exploitation stage*, which was intended to refine the methods and approaches to contextualize the innovations developed by “Lab@AOR” to the daily functioning of healthcare organizations. With the eventual purpose of augmenting the impact of the PPP, the partners decided to adopt an open perspective, engaging institutions operating in contexts other than Italy in the enhancement of the APOTECA ecosystem.

A differentiation approach was taken by the University Hospital of Marche and Loccioni. Entities operating in heterogeneous institutional and cultural contexts were approached for participating in pushing forward the development of the “Lab@AOR”. Starting from 2012 and going on until the new normality ushered by the Covid-19 pandemic, institutions from different countries across the globe have gradually adopted the APOTECA ecosystem (*i.e.*, APOTECAchemo, APOTECAm@a, APOTECAmanager, APOTECAunit, and/or APOTECAps), and have been involved in the APOTECA Community. On the one hand, attention has been focused on expanding the networks to healthcare organizations operating in institutional and cultural contexts which are similar to Italy (*e.g.*, Spain and Germany), with the involvement in the network of the Institut Català d’Oncologia and the Mainz University Hospital. On the other hand, perspectives from different settings have been sought for. The branches of the Cleveland Clinic operating in Ohio (USA) and in Abu Dhabi (UAE) and the Mie University Hospital established in Japan accepted to partake in advancing the innovation ecosystem enacted by “Lab@AOR”. In few years, an international network consisting of 31 knots located in 19 countries and 3 continents arose, which boosted the technological advancements initiated by the introduction of the APOTECA ecosystem.

*Pioneering* and *exploitation* are followed by a *harvesting* stage, during which “Lab@AOR”, in collaboration with the international partners participating to the PPP, made an effort to catalyse the impact of the technological innovations elaborated at the inter-organizational level. Harvesting is realized at three levels. First, it involves the arrangement of reviews and scientific articles which are submitted for publication to scientific journals to increase the visibility of the network and attract new participants. Citing an internal corporate report, “…in 10 years of activity, Lab@AOR has produced 93 scientific publications in national and international journals, 15 academic collaborations, and 23 presentations in conferences”. Second, harvesting is realized through continuous institutional communication, which is intended to augment the institutional legitimacy of the PPP, making it able to establish a better exchange with relevant stakeholders at the international level. Finally, it entails individual and collective training, providing people operating at the organizational level with the knowledge, skills, and competencies required to extract the best advantage from the technological innovations crafted and implemented by the “Lab@AOR”. According to an internal policy document, “…Lab@AOR has trained 487 people, activated 6 education programs, and provided 103.500 education hours”.

The spiral metaphor which has been used to describe the innovation approach adopted by the University Hospital of Marche and Loccioni in activating and nurturing the APOTECA ecosystem can be replicated to describe the growth of the international network enacted by the PPP and the introduction of new projects. *Pioneering*, *exploitation*, and *harvesting* should be understood according to a cyclical perspective. *Harvesting* sets the conditions for a new *pioneering*, which is triggered by the acquisition of distinctive knowledge, skills, and competences obtained from inter-organizational relationships. New *pioneering* which follows the first round of inter-organizational collaboration is inspired by the mission and the vision of the PPP, involving the arrangement of unprecedented technological innovation which are oriented towards patient-centeredness. *Exploitation* is further nurtured by knowledge co-contamination across partners, whose interaction is intended to create new perspectives and insights according to a value co-creation model. This enables partners to advance their capability to thrive in a complex and unpredictable environment, obtaining a unique bundle of resources which is continuously propelled by mutual interorganizational exchanges. Last, but not least, *harvesting* is characterized by the purpose of arranging a new healthcare ecosystem. It enhances the partners’ ability to envision critical technological innovation aimed at improving the wellbeing of people, by putting digitalization and robotization at the service of patient-centeredness.

As an illustrative example of new *pioneering*, the transition towards a “green” hospital was used as a key initiative embodying the idea of community orientation, environment protection, and person-centeredness in the evolution of the “Lab@AOR”. Greenness is related both to the increased sustainability in the accomplishment of conventional organizational activities enacted by technological innovation, and to the creation of a friendly and easy-to-navigate hospital, which enables users, here included people living with limited health literacy, to timely find information needed to access health services. In line with these arguments, new *pioneering* finds a proper expression with the “Green@Hospital” project. It has been undertaken to advance the environmental and social sustainability of healthcare organizations, enabling them to save financial resources by lowering energy consumption and reinvesting them to improve the quality of care in light of a patient-centered perspective. As reported in institutional documents issued by Loccioni, “…in this project “Lab@AOR” coordinated 4 industrial partners, 2 research centres and 4 hospitals across Italy, Greece, and Spain, achieving a 15% energy saving through a gradual digital transformation of hospitals’ operations according to a people-centered perspective”.

## Discussion

Our case study reports the evolution of the partnership between a publicly owned healthcare institution (the University Hospital of Marche) and a private actor (Loccioni), unveiling the dynamics which enabled the former to overcome the major challenges affecting its transition towards automation and robotization. Investigating the formation and development of the PPP, our study fills the knowledge gaps highlighted in previous research about the following steps required to: (1) shift towards Health 4.0 (Cavallone & Palumbo, 2020; Lhotska, 2020), (2) facilitate the achievement of patient-centeredness (Odone, 2021; Palumbo, 2016), and (3) engage multiple stakeholders in the design of the healthcare system of the future (Fürstenau et al., 2019). Furthermore, it gives us some intriguing insights which can be applied outside the healthcare domain. More specifically, the research findings highlight the initial steps forging the build-up of a viable public service ecosystem. It implies a relational model to enhance value in production throughout the process of public services’ provision (Osborne et al., 2021), engaging public and private partners in a collective effort intended to service co-production and value co-creation (Palumbo et al., 2020).

Extant studies have primarily looked at the dyadic relationship between health professionals and patients in the adoption of innovative technologies (Binci et al., 2021). Scholars have tried to shed light on the factors influencing patients’ willingness to accept technologies in the provision of care (Palumbo et al., 2022). Furthermore, attempts have been accomplished to frame the issues faced by health professionals to cope with the digital transformation (Hermes et al., 2020). Conversely, there is limited debate on how PPPs and other forms of collaboration overcome the shortcomings that prevent the digital transition of healthcare institutions by stimulating the development of a knowledge-based service ecosystem. Addressing this knowledge gap, our study presents “Lab@AOR” as an illustrative example of PPP that has been able to gradually involve multiple international players in the journey towards digital transformation and provide an innovative technological solution targeted at enhancing the quality and the appropriateness of care.

We identified three main factors (innovative technology, process analysis, and inter-organizational culture) that underpinned the development of the PPP over an evolution path consisting of three stages (pioneering, exploitation, and harvesting). The introduction and the continuous improvement of a (doomed to be platform) technology represented the starting point of the collaboration between the partners. In undertaking this initial step, the public partner was primarily interested in dealing with a problem related to the automation of hazardous intravenous compounding preparation for oncological patients. Alternatively, the private partner was looking for an ideal setting to assess its robotized technology. The match between public needs and private competences set the ground for the inter-organizational collaboration. It was accomplished through the establishment of transversal teams of professionals, scientists, and experts to address the partners’ mutual expectations and articulate innovative ways to improve health services’ delivery. It is worth noting that the technological solution has been co-created drawing on a deep understanding of the partners’ reciprocal needs. The public actor provided the support to the private company for refining the innovative technology that the latter was already developing, although it missed a testbed to unleash its value.

Process analysis boosted the development of a knowledge-based service ecosystem. Extant research has shown that digital transformation deeply reshapes value creation processes, business models, organizational structures, and management dynamics across different sectors (Lanzolla et al., 2020) and that innovative technologies enable the design of enhanced health services (Kagermann, 2015). Our case emphasizes that, within PPPs, the arrangement of innovative technologies needs to be accompanied by a fully fledged understanding of the processes and procedures characterizing the functioning of the public partner, which hosts the main context within which the collaboration is accomplished. This allows the private partner to collect rich information to refine the technology and spot opportunities that, eventually, lead to continuous improvement and to the attainment of unprecedented innovations. For this to happen, partners should involve their employees in formal and informal inter-organizational meetings and activities, enabling people to work at the crossroads of their organizational boundaries. This empowers employees to factually contribute to the success of the PPP, by adding to value in production implemented at the intersection of the partners (Mugge et al., 2020; Osborne et al., 2022).

Hence, the implementation of the innovative technology fostered by the PPP should be accompanied by the creation of an inter-organizational culture which encourages and sustains individual action towards innovation, promoting the integration of partners’ efforts. This is consistent with the findings of recent studies showing the importance of sticking to a digital culture, having a flexible structure, and being able to react to change in order to adopt innovative technologies in the healthcare context (Binci et al., 2021). It also stresses the need for aligning macro (inter-organizational), meso (organizational), and micro (individual) levels in steering the digital transformation of healthcare institutions (Kokshagina, 2021). In our case, the Green@Hospital project is a spillover generated by the inter-organizational culture established throughout the evolution of the PPP. Beyond providing evidence of the importance of environmental and social sustainability in the healthcare sector (Odone, 2021), the project was the by-product of the curiosity of the University Hospital of Marche, the technical knowledge of Loccioni, and the involvement of employees and stakeholders in a conjoint effort aimed at setting the ground for envisioning the hospital of the future.

The three elements reported above, *i.e.*, innovative technologies, process analysis, and inter-organizational culture, recur in an iterative way in the phases of pioneering, exploiting, and harvesting that depict the evolution of our case over time. The virtuous cycle initiated by the PPP at the local level is currently diffused internationally, witnessing the success of the PPP. Extant studies have found that participation and community building are important practices to design and manage an effective healthcare service platform which is propelled by an inter-organizational effort (Fürstenau et al., 2019). Relying on these arguments, our case exemplifies how community development represents the underlying factor nurturing the digital transformation of healthcare organizations (Cavallone & Palumbo, 2020). This adds interesting insights to scientific evidence claiming that healthcare organizations are moderately ready to undertake a shift towards robotization and automation, but they need a boost to accomplish a smooth digital transition (*e.g.*, Wernhart et al., 2019; Ngusie et al., 2022).

A final remark must be done with respect to the determinants of the PPP’s success. We maintain that three ingredients are combined in the recipe for the viability of “Lab@AOR”. First, the alignment between the public partner’s needs and the private partner’s competencies is crucial. This is clear when considering how the innovative technology has been developed by “Lab@AOR” consistently with the evolving expectations of the public sector partner. Previous studies in innovation management have stressed the importance of inter-organizational fit in the development process of new solutions (Schilling & Hill, 1998). Our results confirm this point, emphasizing the need for achieving a continuous alignment between the partners to conceive impactful innovations. More specifically, our case study suggests that inter-organizational trust is conducive to such an alignment. Formal governance models usually follow and embody spontaneous inter-organizational relationships, which are sedimented in informal exchanges between the partners (Maurya & Srivastava, 2019). Second, the contamination of knowledge permeating all the steps of the collaboration is essential to stimulate the partners’ commitment to the PPP. This is particularly evident during the exploitation and harvesting stages of our case. The multidisciplinary background of members involved in transversal teams allowed the partners to learn from each other, activating a spiralling process of organizational learning and development, which greatly contributed to the advancement of the partnership. Finally, yet importantly, the focus of value generation on the principle of patient-centeredness—which represented the inspiring value of the inter-organizational culture enacted by the PPP—was the glue binding the partners and motivating them to enthusiastically participate in the collaboration.

## Conclusions

Human services are not exempt from the radical transition ushered by the ongoing process of digital transformation. *Inter alia*, healthcare institutions are undergoing a steady process of organizational change, which is targeted towards recontextualizing risk prevention and health promotion services in the cyber-physical domain. However, the institutional and structural complexity of healthcare organizations and their resistance to change slow down their digital turn. Establishing partnership with private sector entities provides publicly owned healthcare organizations with energy to overcome the inertia tying them to conventional models of care, paving the way for a smoother and timelier digital transition and, eventually, transformation. Attempting to shed light on how this dynamic unfolds, the paper provided an example of a PPP which fostered the digital transformation of a publicly owned hospital, while also triggering the development of a knowledge-based public service ecosystem. As detailed below, the study findings inspired both conceptual and practical implications.

### Theoretical implications

One of the main critiques moved against PPP in the healthcare domain is related to their effectiveness (Rajasulochana & Maurya, 2020), although there is evidence of their contribution to public value generation (*e.g.*, Ullah et al., 2012). Drawing on the study findings, we argue that PPPs enable healthcare institutions to overcome the obstacles preventing their digital transformation by virtue of knowledge contamination and integration with consonant private partners (Ma et al., 2021; Ziadlou, 2021). Promoting an alliance between public sector entities and private sector companies to cope with wicked public management issues, PPPs are instrumental to achieve social, environmental, and economic goals, contributing to advancing individual and collective wellbeing (Vecchi et al., 2022). From this standpoint, our case contributes to Osborne et al. (2022) integrative framework on public service ecosystem, delivering an illustrative example of how a PPP (*i.e.*, a particular form of a collaborative governance) inspires the formation of a public service ecosystem, which took its roots in the harvesting and new pioneering stages. The PPP investigated in this research exemplifies how value is co-created for society through co-designed technologies, which enable the community’s access to timelier, personalized and more appropriate health services augmented by robotization and automation.

### Practical implications

From a practitioners’ point of view, our case provides an in-depth description about how to initiate and manage a viable PPP in the healthcare domain. Especially in countries where public expenses in the healthcare industry cannot be expanded due to public budget constraints, we argue that PPPs represent effective solutions to assist the digital transformation of healthcare organizations, supporting them in saving costs and making their processes more effective and efficient, while also advancing technological innovation. Alongside preparing the ground for the healthcare system of the future, PPPs boost the creativity of private actors, enabling them to assess their innovative solution in the healthcare context, a blooming sector from a technological point of view. In sum, PPPs act as preconditions for the establishment and continuous development of technology-based service ecosystems, anticipating future trends in the healthcare domain. Also, they serve as a fertile ground to facilitate knowledge contamination and dissemination across the public sphere and the private realm, which are conducive to a more sustainable and effective health service ecosystem.

### Limitations and avenues for further research

The study findings should be contextualized to the limitations which affected this research. The paper presents a successful case of PPP. Therefore, it might underestimate the dark sides of PPPs, which have been largely reported in the scholarly debate. In addition, although the PPP here investigated expanded internationally in the latest phases of its development, our focus was maintained on the two main partners (*e.g.*, the University Hospital of Marche and Loccioni) throughout the process of data collection and analysis. Hence, we were unable to account for the perspectives and insights of international stakeholders who participated in the PPP. Lastly, although we used multiple sources of information, we did not collect quantitative data highlighting the implications of innovations crafted by the partners on health outcomes. Whilst this constrained the depth of our research, it enabled us to focus our attention on the dynamics of PPP’s establishment and development, consistently with our aim to investigate the steps through which a successful PPP unfolds in the healthcare context.

Acknowledging these limitations permits us to spot avenues for further development. Future research should be addressed at collecting evidence of how partners deal with the side effects of inter-organizational collaborations involving a contamination of the public and the private interest. Attention should be paid to examining the partners’ awareness of the dark sides of PPPs, and their readiness to take preventive actions intended to address the shortcomings generated by the interplay between public sector organizations and private partners. Further research is needed to better understand how successful PPPs are conducive to viable governance models at the meso-level facilitating the establishment of a public service ecosystem. On the one hand, the role of PPPs in nurturing the partners’ commitment to service ecosystems should be analysed. On the other hand, scholars and practitioners should examine the direct and indirect implications of PPPs, analysing how they shape societal norms about standards of public value generation. Last, but not least, research is required to fully disclose how PPPs contribute to the generation of public value, setting the ground for co-creating relationships at the crossroad of the public and the private sectors.

## Data Availability

Data sharing not applicable to this article as no datasets were generated or analysed during the current study.
